# Predicting the Probability of Failure of Cementitious Sewer Pipes Using Stochastic Finite Element Method

**DOI:** 10.3390/ijerph120606641

**Published:** 2015-06-10

**Authors:** Amir M. Alani, Asaad Faramarzi

**Affiliations:** 1 School of Computing and Technology, University of West London, 8^th^ Floor, Villiers House, Ealing Broadway, London W5 2PA, UK; E-Mail: amir.alani@uwl.ac.uk; 2 School of Civil Engineering, University of Birmingham, Edgbaston, Birmingham B15 2TT, UK

**Keywords:** stochastic finite element method, cementitious sewer pipe, probability of failure, concrete corrosion, random variables

## Abstract

In this paper, a stochastic finite element method (SFEM) is employed to investigate the probability of failure of cementitious buried sewer pipes subjected to combined effect of corrosion and stresses. A non-linear time-dependant model is used to determine the extent of concrete corrosion. Using the SFEM, the effects of different random variables, including loads, pipe material, and corrosion on the remaining safe life of the cementitious sewer pipes are explored. A numerical example is presented to demonstrate the merit of the proposed SFEM in evaluating the effects of the contributing parameters upon the probability of failure of cementitious sewer pipes. The developed SFEM offers many advantages over traditional probabilistic techniques since it does not use any empirical equations in order to determine failure of pipes. The results of the SFEM can help the concerning industry (e.g., water companies) to better plan their resources by providing accurate prediction for the remaining safe life of cementitious sewer pipes.

## 1. Introduction

Underground sewer pipes are required to resist and operate safely under various external loads, as well as severe environmental conditions. The degradation of sewer pipes over time in combination with the effects of overlaying soil and surface traffic loads can sometimes cause catastrophic failures in sewer pipes. Despite the ever increasing spending on prevention and mitigation, there are thousands of collapse reports per year in sewer pipe networks throughout the world. The consequences of collapses of sewers are socially, economically, and environmentally devastating, causing, e.g., enormous disruption of daily life, massive costs, widespread pollution, and so on. It is known that sewer collapses are predominantly caused by deterioration of the pipes. For cementitious sewer pipes, corrosion is the main cause of deterioration. Corrosion can cause reduction in structural strength of the pipeline, leading to pipe collapse. Severe localised corrosion can cause pitting; also resulting in the failure of sewer pipes [1,2,3,4]. Therefore, considering the effect of corrosion in the analysis and design of cementitious sewer pipes is an essential element in developing an advanced model to predict the likelihood of collapses of sewer systems. Modelling corrosion in the structural analysis of a pipeline and simulating the performance of the pipe under various external load conditions together with the effect of corrosion provide a practical tool for both designer and asset managers to predict the service life or remaining safe life of the system.

A number of research studies in literature are devoted to the probabilistic analysis and reliability of underground pipelines. Ahammed and Melchers [5] derived a formula to estimate the maximum circumferential stress in underground, pressurised pipes subjected to external loading and corrosion. They combined a nonlinear time-dependant function for modelling the corrosion, and empirical equations proposed by Marston and Spangler [6,7] to estimate circumferential stresses in pipes due to the soil self-weight and traffic load. Then, using the obtained formula, they performed a reliability analysis to evaluate the probability of the failure of sewer pipes *versus* their service life. Ahammed and Melchers [8] extended their work to evaluate the reliability of underground, pressurised pipes, subjected to both circumferential and longitudinal stresses and corrosion. The equations developed by Marston and Spangler and used by Ahammed and Melchers [5,8] can provide estimations for stresses in the pipes due to external loads. However, these equations contain a number of empirical coefficients, which are usually estimated on the basis of limited information. The accuracy of the selected coefficients can significantly affect the accuracy of the predicted stresses in the pipe. Tanaka *et al.* [9] presented a mathematical model to find the survival probability of underground water pipelines. They demonstrated the application of the model to the water distribution network of Osaka, Japan. They also used the model to find the optimum renewal interval time for the different types of pipes in the network. Li *et al.* [10] used the Monte Carlo simulation technique to calculate the remaining safe life of metallic underground gas pipelines. They performed a sensitivity analysis to identify the key corrosion parameters in terms of pipeline failure. Lee *et al.* [11] presented the application of probabilistic modelling to evaluate the long-term performance of pipes. They evaluated the reliability of deteriorated pipes that were subjected to internal fluid pressure, external soil pressure and traffic loading. These studies and many similar works within literature have used empirical equations to estimate the stresses in the pressurised pipes. Alternative approaches, based on robust theoretical and numerical methods, are required to provide a more accurate estimation of the stresses in the pipes due to external loading and corrosion. In addition, there is a lack of research work in the area of reliability of cementitious sewers. It is acknowledged that H2S-induced (Hydrogen Sulphide) corrosion for cementitious pipes may have been well-researched but little is known about the combined effects of corrosion and external pressures on their remaining safe life in a quantitative manner. This may lead to a situation whereby some pipes may be replaced prematurely whilst others should be replaced but have not been. The former obviously is not cost-effective and the latter potentially presents undesirable challenge with devastating consequences. The developed model in this paper is an attempt to effectively eliminate this situation by accurately predicting pipes’ remaining safe life, and ultimately contributing towards a more effective tool for planning. 

In order to provide an accurate prediction of the remaining safe life of the sewer pipes, the parameters that affect and control the process of deterioration and failure of pipes, the interaction of different mechanisms of failure, and their effect on the remaining safe life of sewer pipes should be considered. Due to the large degree of uncertainty relating to the factors that are involved in the operation of underground sewer systems––in particular corrosion––it is probably more rational to model the failure of sewer pipes as a stochastic process. To fulfil this, a model has been developed and is reported in this paper that predicts the failure of cementitious sewer pipes using a stochastic finite element method. The developed stochastic finite element model can determine the prospect of failure of sewer pipes throughout their intended service life. Particular attention is paid to simulate the corrosion using the stochastic finite element model and to investigate its interaction with other mechanisms of failure and their effect on the remaining safe life of sewer pipes. Moreover, using the developed stochastic finite element model a parametric study is carried out to evaluate the significance and effect of each contributing parameter on the remaining safe life of the cementitious sewer pipes. The results provided by the developed stochastic finite element model can help asset managers and owners to make risk-informed and cost-minimised decisions with respect to when, where, what, and how interventions are required to ensure the safety and integrity of existing pipelines during their whole life of service.

## 2. Stochastic Finite Element Method 

Stochastic finite element method (SFEM) is a powerful numerical tool in computational stochastic mechanics. SFEM can be classified as an extension of the classic deterministic finite element approach to the stochastic framework, *i.e.*, to the solution of static and dynamic problems with stochastic mechanical, geometric, and/or loading properties [12].

The finite element method is a numerical technique used to obtain solutions to a wide range of partial differential equations describing various engineering problems including aerospace, geotechnical, structural engineering and many others. Most of the problems in engineering design and analysis can be modelled as a single or a set of differential equations. These differential equations describe the response of a system subjected to external influences. Many differential equations cannot be solved analytically and usually numerical techniques are used to find their approximate solutions. Of these numerical techniques the finite element method is known to be one of the most powerful general techniques for the numerical solution of a variety of problems encountered in engineering. The basic idea behind the finite element method is to divide the structure, body, or region being analysed into a large number of elements [13]. For a linear static analysis of a system the finite element method results in the following system of equations:

**KU = F** (1)

where **K** is the global stiffness matrix and **U** and **F** represent the nodal displacement and force vectors. The global stiffness matrix (**K**) is obtained by adding the stiffness matrices of all elements,

(2) $$K=\sum K^{e}$$

where **K^e^** is:

(3) $$K^{e}=\int_{\Omega }B^{T}DB\;d\Omega$$

In the above equation **B** is the strain matrix, **D** is the elasticity tensor, and Ω is elemental domain. The system of equations is solved to obtain the nodal displacements **U** and eventually other parameters such as stresses, strains *etc.* When one or some of the variables of the model (*i.e.*, the loading and/or material properties and geometries) involve uncertainty, Equation (1) can be written in the following form [14,15]:

**K**(ω)**U**(ω) **= F**(ω) (4)

where ω represents the randomness of the parameters. The above equation is a stochastic representation of the static finite element problems and the uncertain response of structure (*i.e.*, **U**(ω)) and other quantities of interest such as stresses **σ**(ω), and strains **ε**(ω) can be obtained by solving Equation (4).

Although there are a number of variants of SFEM in the literature, it has been proven that Monte Carlo simulation (MCS) SFEM is by far the most general and powerful method to estimate the uncertainty of a structure’s response [12,14,15,16,17,18,19]. In the MCS-based SFEM, a deterministic finite element problem is solved a large number of times and the response variability is calculated using statistical relationships. The MCS method does not involve any simplification or assumption, which makes it a robust and universal technique to treat complex SFEM problems. The only difficulty of MCS could be the significant computational cost that is required to perform a large number of simulations particularly when analysing problems with low probabilities of failure. However the recent advances and improvements in computer hardware and the use of efficient sampling methods to reduce the number of simulations, such as importance sampling [20], subset simulation [21], line sampling [22], local averaging [23,24], Latin-hypercube [25], and many more, have enabled the MCS-based SFEM to become a strong tool in the performance of the probabilistic analysis of various finite element models with uncertainty in load, displacement, and material properties.

MCS-based SFEM involves the generation of random numbers (random variables) with a uniform distribution between 0 and 1. Then the inverse cumulative probability function of the random numbers is used to produce the random numbers with arbitrary distribution function. The finite element model is created using both the deterministic parameters and the generated random variables. Limit state function(s) *Z*(**X**) of the problem is defined such that *Z*(**X**) ≤ 0 shows failure and positive values of *Z*(**X**) indicate no failure region. At every simulation, the limit state function(s) is checked using the finite element method (e.g., if the resultant stress has exceeded the yield stress) and the probability of failure is obtained using the following equation [20]:

(5) $$P_{f}^{i}\;=\;\frac{N_{f}}{N}$$

where $P_{f}^{i}$ is defined as the probability of failure of each limit state function, *N_f_* is the number of simulations when *Z*(**X**) ≤ 0, and *N* is the total number of simulations. In a series system, where more than one limit state function exists, the failure of any of the limit state functions implies the failure of the system. If the individual failures are mutually independent, then the probability of the system can be obtained from:

(6) $$P_{f}=1-\prod_{i=1}^{m}\left(1-P_{f}^{i}\right)$$

where *P_f_* is the probability of failure of the system, and is the number of limit state functions defined for the system.

In this paper an in-house SFEM code based on the integration of the finite element method and Monte Carlo simulation technique is developed to analyse the probability of failure of underground cementitious sewer pipes. The finite element code is equipped with a library of different 2D and 3D elements and a relatively fast solver to handle large size meshes. In the MCS code, the Latin-hypercube sampling technique is used to generate the random numbers with arbitrary inverse cumulative probability functions. In addition, the developed code is employed incrementally over the time in order to account for the degradation of the sewer pipe and predict the probability of failure of the sewer pipes throughout their service life.

In the SFEM, each uncertain parameter is represented by a random variable with a known probability density function, a mean value and a coefficient of variation. The random variables together with the deterministic parameters are used to generate the input file of the finite element model. The finite element analysis is performed and at the end of each simulation the failure criteria are checked. The failure criteria adopted in this study for the reliability analysis of cementitious sewer pipes are (i) the loss of strength of pipe; a Drucker-Prager yield criterion has been adopted to check if the yield strength of the pipe material has been reached, and (ii) excessive reduction of the pipe wall thickness due to the corrosion; the amount of corrosion is checked at every simulation step against a minimum allowable pipe wall thickness (this is explained in further detail in the next section). Each failure criterion is expressed as a limit state function and the probability of failure of the whole system is calculated using Equation (6).

## 3. Effect of Corrosion

Concrete corrosion due to sulphuric acid attack is known to be one of the main contributory factors in the degradation of concrete sewer pipes. Sulphate, which exists in wastewater, is reduced to sulphide by anaerobic bacteria. These bacteria are present in a thin slime layer on the submerged surface of the sewer pipe and the production of sulphide occurs in this slime layer. The generated sulphide escapes to the exposed sewer atmosphere where it is transformed to sulphuric acid by aerobic bacteria. The acid reacts with the cementitious sewer pipe, which forms gypsum and causes corrosion [26,27,28]. Pomeroy [27] proposed an equation to predict the corrosion rate in sewer pipes. The proposed equation by Pomeroy [27] did not take into account the effect of the age of the pipe on corrosion rate. However a brief study of the available data on the corrosion of cementitious sewer pipes in literature can confirm that the loss of wall thickness does not occur at a constant rate over the design life of the pipe [29]. Therefore, in order to tackle this issue, in this study it is assumed that the corrosion of concrete sewer pipes is dependent on the age of the pipe and can be modelled in a practical engineering way to account for a time dependant corrosion process using a power law model. This approach has also been used by other research studies to model the corrosion of underground water and gas pipelines [5,8,30]. The power law model to predict the biogenic sulphuric acid corrosion of concrete pipes can be presented in the form of the following equation:

*C =* α*T*^λ^ (7)

where *C* is the corrosion of the pipe, α is a multiplying coefficient, λ is an exponential coefficient, and *T* is the age of the pipe. The data provided in Meyer [29] are utilised to evaluate the coefficients in Equation (7) using an exponential regression. Due to a large degree of uncertainty relating to the corrosion process, the corrosion and consequently the coefficients in Equation (7) are considered as stochastic parameters.

**Figure 1:**
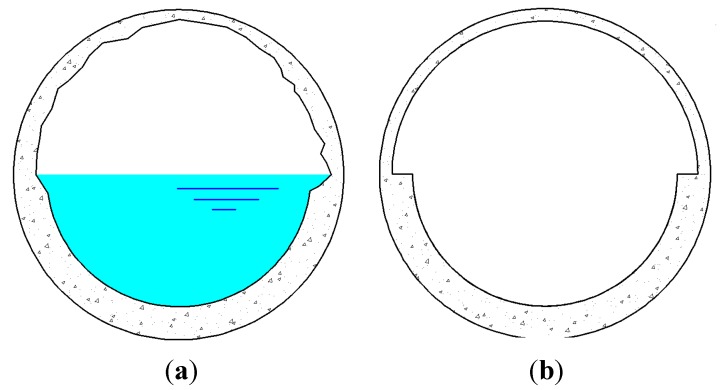
Corrosion in concrete sewer pipes (**a**) Realistic distribution of corrosion in a concrete sewer pipe (non-uniform); (**b**) Idealised corrosion of concrete sewer pipes in the SFEM (uniform).

The predictions provided by Equation (7) are used in two parts of the simulation process. First, they are used to check whether the corrosion limit state function is violated or not. If the value provided by Equation (7) has exceeded the minimum allowable wall thickness then it is recorded as a corrosion failure and will be used to ascertain the probability of failure due to corrosion (Equation (5)). Equation (7) is also used to update the finite element mesh of the pipe at every simulation to represent the reduction of the wall thickness due to corrosion. It is worth mentioning that corrosion only attacks the exposed surface of the sewer pipe, above the sewage, with its deepest penetration in the crown of the pipe [27]. Although the actual distribution of the corrosion in a concrete sewer pipe is unequal (Figure 1a), in this study it is assumed that the corrosion has a uniform distribution (Figure 1b). For every finite element simulation the amount of corrosion is obtained using Equation (7) and the finite element input file is updated by re-meshing the pipe domain using the new coordinates after considering the corrosion. The strength of the concrete pipe is expected to be reduced as a result of the reduction of pipe wall thickness.

## 4. Numerical Example

**Figure 2:**
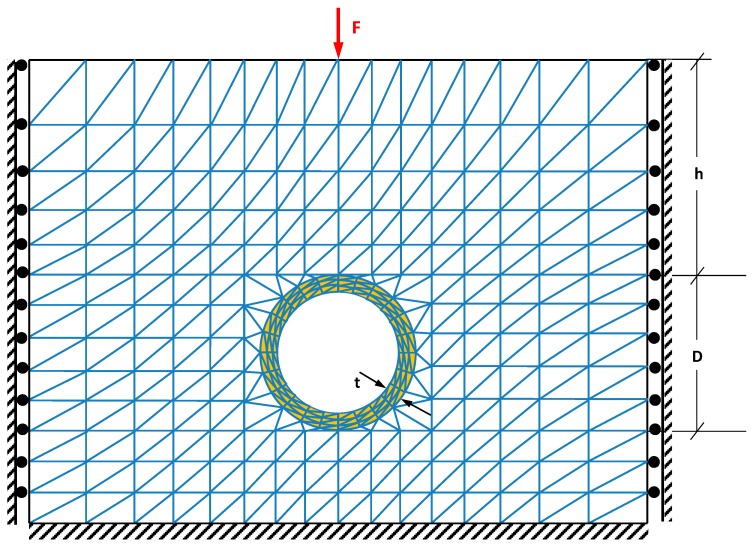
Geometry, mesh and parameters used in the FE model.

In this section a numerical example is considered to evaluate the performance of the developed SFEM in predicting the probability of failure of concrete sewer pipes subjected to stresses and corrosion. The presented example is also used to investigate the influence of the contributing parameters in the remaining safe life of the sewer pipes and their interaction with each other. The finite element (FE) model of the problem consists of a concrete pipe with a circular section buried underground and surrounded by a homogenous soil. The model is subjected to self-weight and an external traffic load applied on the surface of the model. The FE model is assumed to be two dimensional with plane strain geometrical condition (*i.e.*, 2D plane strain elements are used to create the FE mesh). In order to draw conclusions that are not affected by a particular example, the problem is scaled with respect to the external diameter of the pipe and the variations of different normalised parameters are investigated. Figure 2 shows the geometry, boundary conditions, and a typical FE mesh of the problem.

The normalised parameters are expressed in Equations (8–10).

(8) $$h^{*}\;=\;\frac{h}{D}$$

(9) $$t^{*}\;=\;\frac{t}{D}$$

(10) $$F^{*}\;=\;\frac{F}{\gamma \;h\;D^{2}}$$

In the above equations, *h* is the height of the soil under which the pipe is buried, *t* is the initial thickness of pipe, *D* is the external diameter of the pipe, *F* is the traffic wheel load, and γ is the unit weight of the soil.

In addition to the deterministic parameters such as diameter and thickness of the pipe, there are some parameters that are considered as stochastic or random. Using the existing studies on the reliability analyses of underground pipelines (e.g., [5,8]) and, after performing a number of pilot simulations, five parameters were chosen as random variables. These parameters, their mean and their coefficient of variation (cv) are presented in Table 1. The time-dependant growth of the traffic load over the lifetime of the pipe has been ignored in this study. Other parameters, such as unit weight of soils, were considered deterministic mainly because their coefficients of variation were very insignificant or they had small or negligible effects on the probability of failure of the system.

**Table 1:** Random variables used for the SFEM.

Symbol	Description	Mean	Coefficient of Variation
*f_c_*	Concrete maximum compressive strength	35 MPa	0.1
F*	Traffic Load	Varies from 10–20	0.25
*I_f_*	Load impact factor	1.5	0.15
α	Corrosion multiplying coefficient	3.5 × 10^−5^	0.1
λ	Corrosion exponential coefficient	1.5	0.15

## 5. Results and Discussion

**Figure 3:**
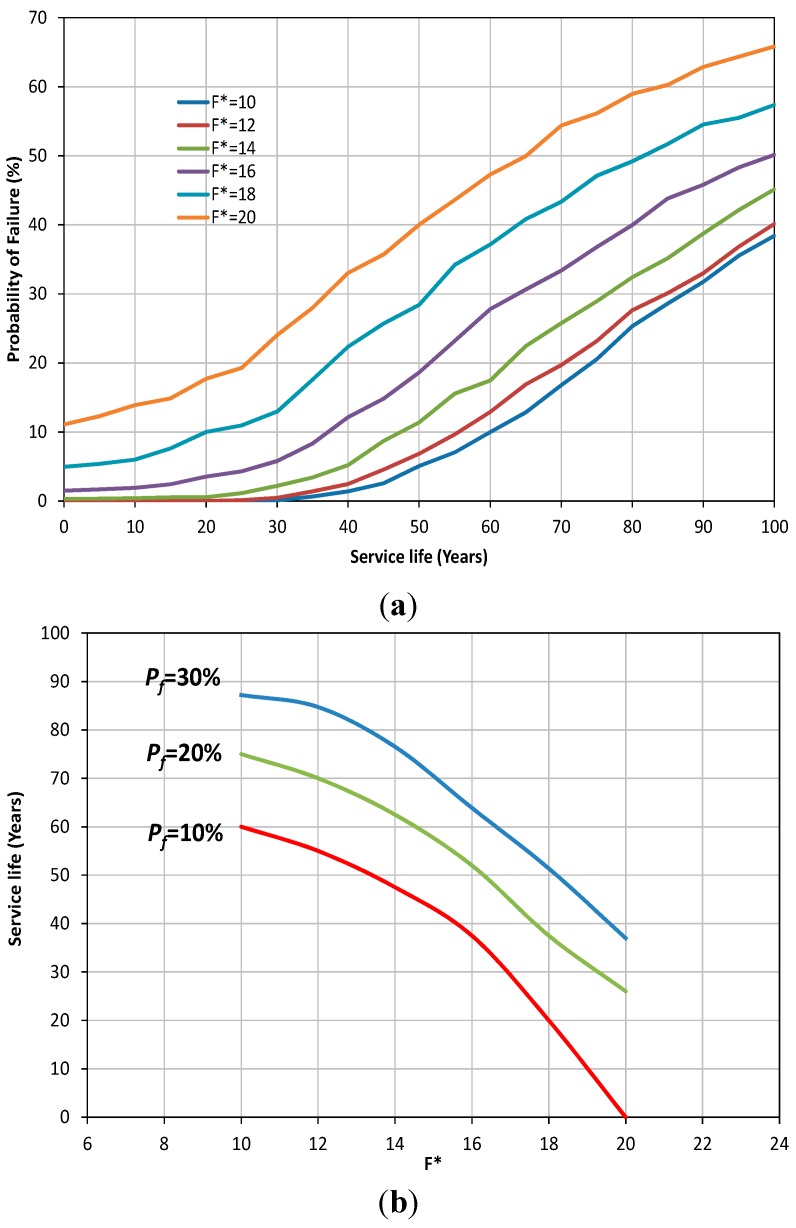
(**a**) Probability of failure of concrete sewer pipe for different values of scaled traffic load *versus* service-life; (**b**) The variation of traffic load (normalised) with the service life of sewer pipes.

After choosing the type of each parameter (deterministic or stochastic) and creating the FE models, the time-dependant SFEM were performed for different values of scaled parameters in order to study their effect on the probability of failure of concrete sewer pipes. Figure 3a shows the results of the SFEM on probability of failure of the example sewer pipe under various normalised traffic loads over its service life. It can be seen that, as the traffic load increases, the probability of failure of sewer pipes grows rapidly. In addition it can be noted that initially the probability of failure is zero or very small for all cases; however as the effect of corrosion emerges (usually after the first 20 years) the probability of failure increases rapidly. To further investigate the effect of traffic load on the service life of concrete sewer pipes subjected to stresses and corrosion, the following analysis was also carried out. Let us assume that the acceptable probability of failure (*P_f_*) is 10% (equivalent of a remaining safe life of 90%). The service life of each FE model (each model has a different normalised traffic load) can be evaluated using the results provided by the SFEM (Figure 3b). It can be seen in Figure 3b that the service life of the sewer pipe is reduced significantly with a non-linear trend as the traffic load increases. For example if the traffic load is doubled (*i.e.*, F* increases from 10 to 20) then the service life of the sewer pipe is reduced from 60 years to zero years for the acceptable probability of failure of 10%. A similar trend can also be seen for other presumed acceptable values of probability of failure (*i.e.*, *P_f_* = 20% and *P_f_* = 30%).

**Figure 4:**
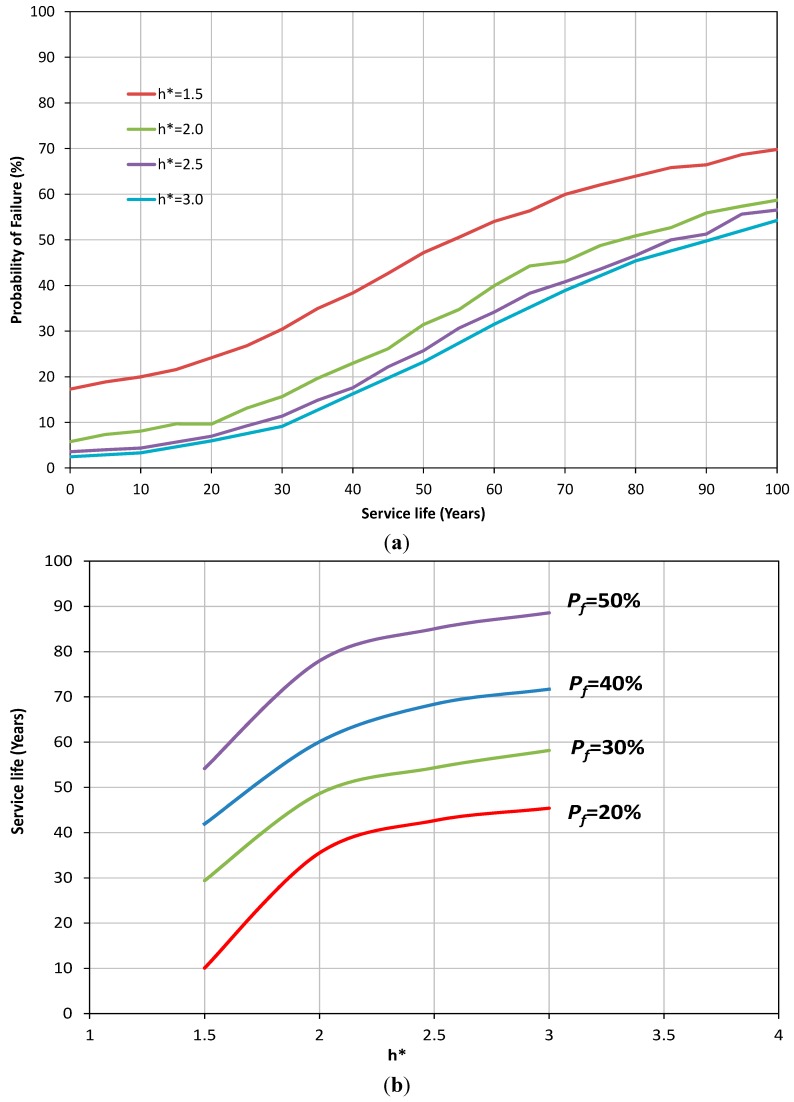
(**a**) Probability of failure of concrete sewer pipe for different values of h*; (**b**) The variation of service life of sewer pipe *versus* different h*.

**Figure 5:**
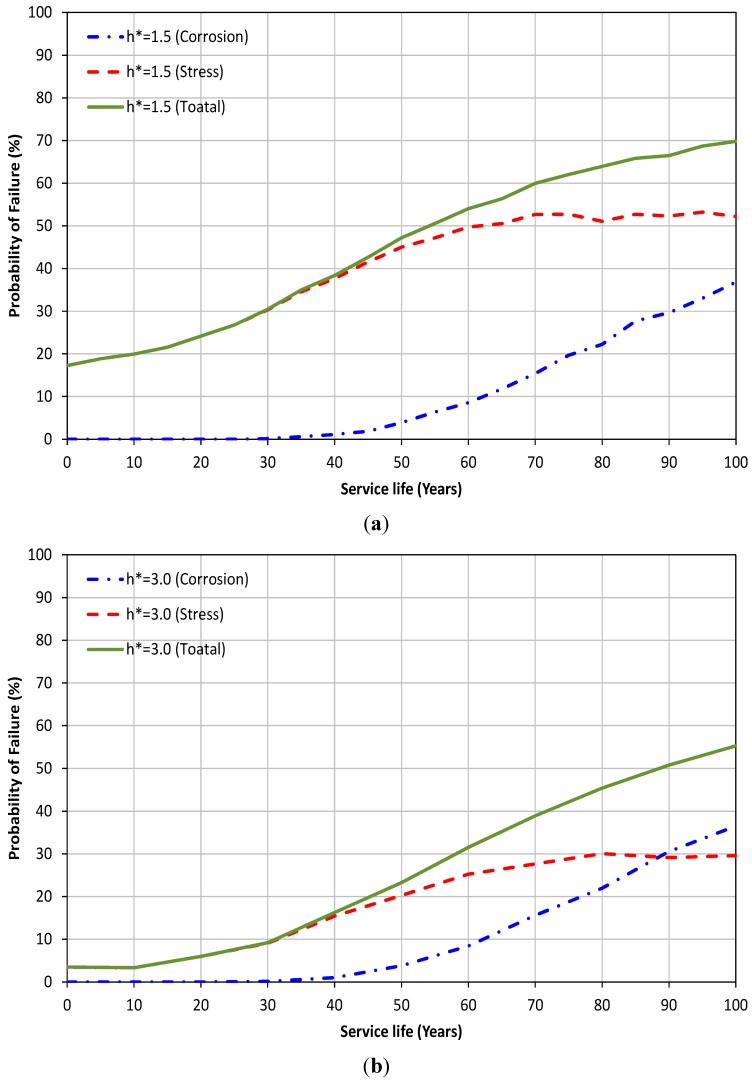
Probability of failure due to corrosion, stress and total failure for (**a**) h* = 1.5; (**b**) h* = 3.0.

**Figure 6:**
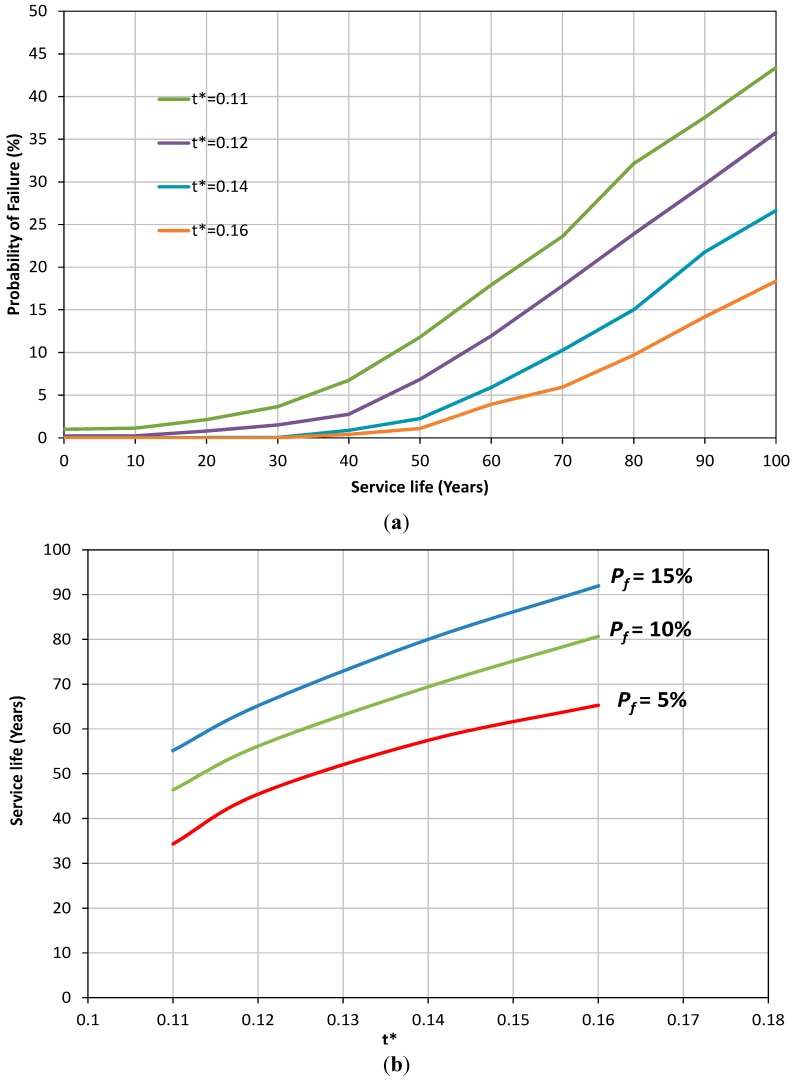
(**a**) Probability of failure of concrete sewer pipe *versus* its service life for different values of normalised pipe thickness; (**b**) The variation of service life of sewer pipe *versus* different t*.

The effect of the buried depth of the sewer pipe on the probability of failure is shown in Figure 4a. The figure shows that reduction in soil cover can result in an increase in the probability of failure of sewer pipes with a nonlinear relationship. In Figure 4b, it is shown that an increase of 100% of soil cover height can extend the service life of the sewer pipe by at least an extra 30 years. The reduction in probability of failure of sewer pipes for higher values of h* can be attributed to the fact that at greater depths, the effect of traffic load becomes smaller compared to the corrosion, particularly as the age of the pipe increases. This results in a situation where the corrosion failure may become the main mechanism of failure. As can be noted from Equation (7), corrosion is not a function of depth and the same values are applicable for both deep and shallow pipes. Further details can be seen in Figure 5. In Figure 5 the probability of failure due to corrosion, stress and total failure are presented for two different depths. It can be noted that the main cause of the failure of sewer pipe for shallow depth, h* = 1.5 (Figure 5a), is the stress throughout the service life of the pipe while for h* = 3.0 (Figure 5b) corrosion becomes the dominant mechanism of failure after 90 years of service life. Similar conclusions to those obtained for the effect of traffic load and cover soil on probability of failure can be drawn for the thickness of the pipe (see Figure 6a,b).

**Figure 7:**
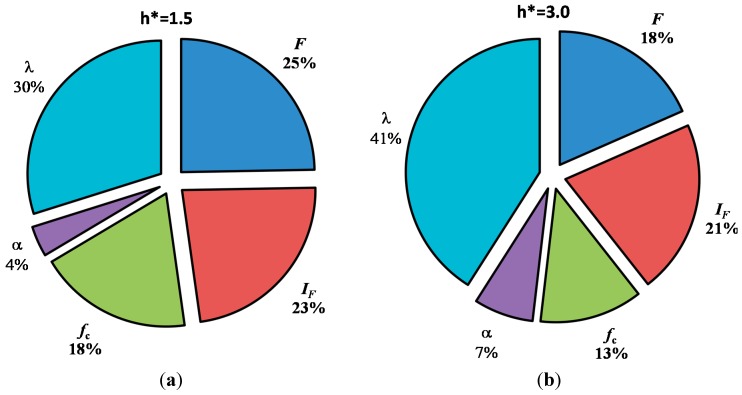
Relative contribution of random variables to the probability of failure of sewer pipe after 100 years of service life (**a**) h* = 1.5; (**b**) h* = 3.0.

Figure 3, Figure 4, Figure 5 and Figure 6 show the effect of both deterministic (*i.e.*, h* and t*) and stochastic (F*) parameters on the probability of the failure of sewer pipes. In order to investigate the relative contribution and importance of each stochastic (random) variable in the probability of failure of concrete sewer pipes, a sensitivity analysis was carried out. The results of the sensitivity analyses for two examples with normalised soil cover (h*) after 100 years of service life are presented in Figure 7a,b. In both figures it can be seen that the exponential coefficient of corrosion extensively contributes to the probability of the failure of concrete sewer pipes. It can also be noted that this contribution enlarges as the soil cover increases. As previously explained, this is mainly due to the fact that, at higher values of soil cover, the dominant failure mode can become the corrosion failure as the pipe passes a certain age. A further point to discuss is the importance of the models that predict the corrosion in sewer pipes. For example, at a scaled buried depth of h* = 3.0 it can be seen that the corrosion coefficients (*i.e*., α, λ) together contribute to nearly half (48%) of the probability of failure of sewer pipes. This shows the significant influence of corrosion in general and corrosion coefficients in particular on the prediction of probability of failure. In general, the results of the sensitivity analysis can be used to efficiently improve the design towards a higher reliability for sewer pipe projects or to enhance management, rehabilitation and spending on existing pipelines.

## 6. Summary and Conclusions 

In the present study a stochastic finite element method was utilised to predict the probability of failure of concrete sewer pipes subjected to combined effects of corrosion and stresses. Uncertainties involved in pipe material, traffic load, and corrosion are considered to develop the stochastic finite element model. A nonlinear time-dependent model was chosen to predict the corrosion in concrete sewer pipes. The model parameters were chosen based on a set of existing data in the literature. A normalised numerical example was employed to investigate the effect of both deterministic and probabilistic parameters on the probability of failure of sewer pipes. Two mechanisms of failure (*i.e.*, corrosion and stress failure) were adopted to define the limit state functions. The results of the numerical simulations revealed a nonlinear relationship between most of the parameters and the probability of failure of sewer pipes. In addition the results of the sensitivity analyses showed the significant contribution of the corrosion parameters. Study of the available literature clearly demonstrated that there is a serious lack of monitoring data of underground sewer pipes. The monitoring field data compiled by water industry are neither readily available nor comprehensive enough to be utilised for appropriate analytical purposes. No doubt access to such data is of paramount importance to check the validity of developed model in this study. The authors are currently working with concerning industries in order to have access or compile the much-needed data. 

The results of the SFEM can be used to improve the performance and planning of existing sewer systems, by providing better predictions for the probability of failure of sewer pipes compared to the existing approaches. The SFEM can bring together the effects of contributing parameters in the probability of failure of the system being studied in a numerical framework with high precision. Using the SFEM, it is possible to study the effect of each parameter on the failure of the system and their interaction with each other. The SFEM also provides a time-dependant reliability analysis for predicting the remaining safe life of sewers, which provides a means to better manage the existing sewers and plan resources during their whole life of service.
